# An analysis of DNA methylation in human adipose tissue reveals differential modification of obesity genes before and after gastric bypass and weight loss

**DOI:** 10.1186/s13059-014-0569-x

**Published:** 2015-01-22

**Authors:** Miles C Benton, Alice Johnstone, David Eccles, Brennan Harmon, Mark T Hayes, Rod A Lea, Lyn Griffiths, Eric P Hoffman, Richard S Stubbs, Donia Macartney-Coxson

**Affiliations:** Biomarkers Group, Environmental Health, Institute of Environmental Science and Research (ESR), Wellington, 5022 New Zealand; Genomics Research Centre, Institute of Health and Biomedical Innovation, Queensland University of Technology, Kelvin Grove, QLD 4059 Australia; The Wakefield Clinic, Wellington, 6242 New Zealand; Integrative Systems Biology, George Washington University School of Medicine and Health Sciences, Washington, DC 20010 USA; The Wakefield Biomedical Unit, Department of Pathology and Molecular Medicine, University of Otago, Wellington, New Zealand

## Abstract

**Background:**

Environmental factors can influence obesity by epigenetic mechanisms. Adipose tissue plays a key role in obesity-related metabolic dysfunction, and gastric bypass provides a model to investigate obesity and weight loss in humans.

**Results:**

Here, we investigate DNA methylation in adipose tissue from obese women before and after gastric bypass and significant weight loss. In total, 485,577 CpG sites were profiled in matched, before and after weight loss, subcutaneous and omental adipose tissue. A paired analysis revealed significant differential methylation in omental and subcutaneous adipose tissue. A greater proportion of CpGs are hypermethylated before weight loss and increased methylation is observed in the 3′ untranslated region and gene bodies relative to promoter regions. Differential methylation is found within genes associated with obesity, epigenetic regulation and development, such as *CETP*, *FOXP2*, *HDAC4*, *DNMT3B*, *KCNQ1* and *HOX* clusters. We identify robust correlations between changes in methylation and clinical trait, including associations between fasting glucose and *HDAC4*, *SLC37A3* and *DENND1C* in subcutaneous adipose. Genes investigated with differential promoter methylation all show significantly different levels of mRNA before and after gastric bypass.

**Conclusions:**

This is the first study reporting global DNA methylation profiling of adipose tissue before and after gastric bypass and associated weight loss. It provides a strong basis for future work and offers additional evidence for the role of DNA methylation of adipose tissue in obesity.

**Electronic supplementary material:**

The online version of this article (doi:10.1186/s13059-014-0569-x) contains supplementary material, which is available to authorized users.

## Background

Obesity is a major public health problem and a risk factor for type-2 diabetes, hypertension and cardiovascular disease [1-4]. Adipose tissue is an endocrine organ [5] and plays a fundamental role in obesity-related metabolic dysfunction [6]. Different adipose tissue depots within the body have distinct structural and biochemical properties [7,8]. In humans, these depots can broadly be divided into two categories, subcutaneous and intra-abdominal. Metabolic risk is affected by both the distribution of body fat between different adipose tissue depots within an individual and the differing natures of the depots themselves [9-11]. Increased intra-abdominal adipose tissue has been correlated with an increased risk of obesity-related co-morbidities such as cardiovascular disease, atherosclerosis and type-2 diabetes [12-14].

Causal genetic variants of obesity and type-2 diabetes have been identified through candidate gene, family-based linkage and large scale association analyses. However, apart from the small number of rare monogenic disease variants application of genetics to clinical management has not occurred. Reasons for this include the relatively small effect size of common genetic variants, the location of a large number of disease associated loci in intergenic regions of the genome, and the likelihood that additional causal variants are yet to be identified [15,16].

It is now recognised that epigenetics plays a significant role in complex disease, and provides mechanisms whereby environmental factors can influence complex diseases such as obesity and type-2 diabetes [17,18]. Thus, epigenetics may explain some of the ‘missing heritability’ of complex disease, and, because of effects on regulation, provide a functional role for some of the intergenic loci associated with disease.

Animal and human studies have shown that the DNA methylation status of genes in offspring can be altered *in utero* by the maternal dietary environment [19-23]. Godfrey *et al.* [24] further reported that the child’s methylation status correlated with adiposity in later life. Feinberg *et al.* [25] identified four variably methylated regions in lymphocytes which correlated with body mass index (BMI); these regions were located in or near genes previously implicated in body weight regulation or diabetes. Obesity has also been associated with genome-wide DNA methylation changes in peripheral blood [26-28], and methylation differences have been reported in peripheral blood in response to caloric restriction [29] and weight loss intervention [30]. In addition, a recent genome-wide analysis of peripheral blood revealed type-2 diabetes related DNA methylation variations [31], and another investigated associations between methylation and fasting glucose, insulin and insulin resistance [32].

Epigenetic signatures are acquired by cells during development and differentiation. DNA methylation changes, such as demethylation of specific sequences within the *leptin* and *GLUT4* gene promoters is observed during adipogenesis [33,34] and adipose tissue precursor cells retain their DNA methylation profile through generations of culture [35]. In addition, women with a lower baseline methylation of the *leptin* and *TNF-alpha* gene promoters in subcutaneous adipose tissue responded better to dietary intervention [36].

A small number of studies have reported DNA methylation profiling of human subcutaneous adipose tissue. The first, by Bouchard *et al.* [37] assayed 15 K CpG dinucleotides (CpGs) in subcutaneous adipose tissue from high (3% to 6% loss of body fat) and low (≤3% loss of body fat) responders to caloric restriction reporting DNA methylation differences between the two groups at 35 loci before weight loss and 3 loci after weight loss. More recently a couple of studies have investigated DNA methylation differences between twins [38,39] and another compared thigh subcutaneous adipose before and after exercise intervention [40]. Investigation of the omentum DNA methylome has been reported in the context of both an identification of tissue-specific differentially methylation using the Illumina 450 K platform [41], and a comparison of global methylation patterns with subcutaneous adipose using a luminometric assay [42].

Given this increasing evidence for the involvement of epigenetics in complex disease and obesity in particular [17,18], together with the role that adipose tissue plays in disease progression, we hypothesised that epigenetic changes would be present in this tissue in the context of obesity and weight loss. Roux en Y gastric bypass surgery (from now on referred to as gastric bypass) is used to treat morbid obesity and results in marked weight loss [43,44]. Therefore, we use gastric bypass as a model for significant weight loss. Two studies have investigated global DNA methylation before and after gastric bypass, one in skeletal muscle and the other in liver [45,46]. Here we extend this to two adipose tissue depots and present the first high density DNA methylation profile of subcutaneous abdominal and intra-abdominal omental adipose tissue before and after gastric bypass and weight loss.

## Results

### Differential methylation of adipose tissue before and after weight loss

#### Global differential methylation

**Figure 1:**
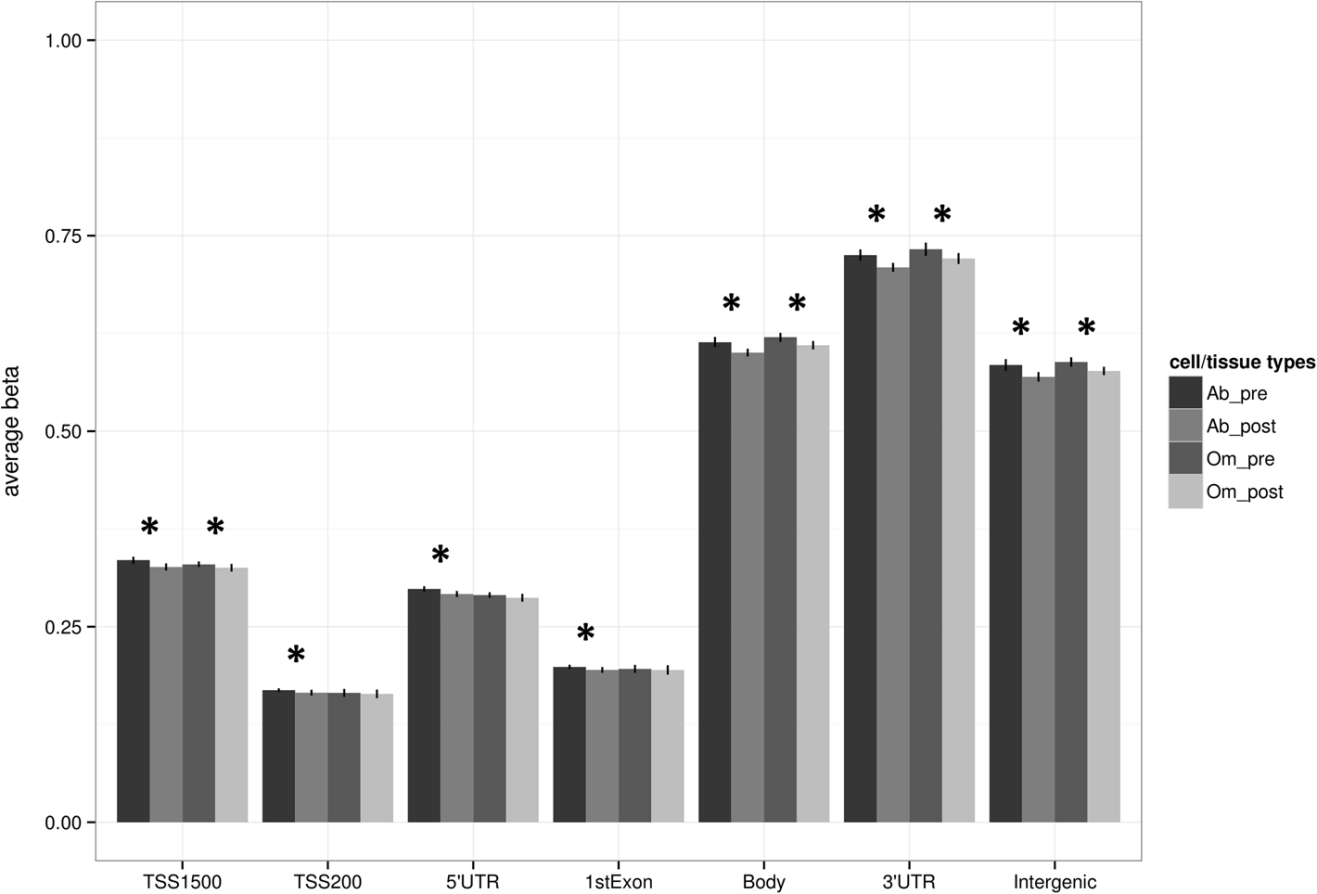
**Global DNA methylation in human adipose tissue plotted by gene region.** Gene regions are based on Illumina 450 K beadchip annotation. Data are presented as mean +/− standard deviation. *Significant difference between average DNA methylation before and after weight loss, Bonferroni adjusted *P* <0.05. TSS proximal promoter defined as 200 bp (TSS200) or 1,500 bp (TSS1500) upstream of the transcription start site. UTR: untranslated region. Ab – subcutaneous adipose, Om – omentum before (pre) and after (post) gastric bypass and weight-loss.

DNA methylation was analysed independently in subcutaneous abdominal adipose and intra-abdominal omental adipose (referred to as subcutaneous adipose and omentum, respectively, from this point) before and after gastric bypass and weight loss in 15 women (clinical and anthropometric data presented in Table 1). We investigated global DNA methylation (450,315 CpG sites passing quality filtering) and plotted this at the level of genomic gene annotation (Figure 1). This revealed a clear pattern of methylation across the genome with least methylation in promoter regions and greatest methylation in the 3′ UTRs (untranslated regions) and gene bodies. While the overall pattern was the same in both adipose tissues, significantly more methylation was observed before than after weight loss for all gene regions in subcutaneous adipose and all except TSS200, 5′UTR and the first exon in omentum (Figure 1). In order to investigate if the global pattern of methylation across gene regions was similar in other tissues we investigated publically available data from 15 different tissues (including two samples from healthy normal adipose). We observed a very similar pattern of global DNA methylation across all tissue samples analysed (Additional file 1).

**Table 1:** **Clinical and anthropometric data for individuals in the study**

**Trait**	**Before weight loss**	**After weight loss**	**T statistic**	***P*** **value**
Age (years)	44 (+/− 10)	45 (+/− 10)	−7.64	1.16 × 10^−6^
Weight (kg)	123.9 (+/− 33.32)	76.2 (+/− 15.4)	8.31	4.42 × 10^−7^
BMI (kg/m^2^)	47.6 (+/− 11.3)	29.3 (+/− 5.5)	9.10	1.48 × 10^−7^
Glucose (mmol/L)	5.5 (+/− 1.2)	4.6 (+/− 0.6)	2.35	0.017
Insulin (pmol/L)	176.2 (+/− 168.1)	33.4 (+/− 19.3)	3.04	0.006
HbA1c	6.0 (+/− 0.6)	5.5 (+/− 0.5)	2.42	0.018
Triglycerides (mmol/L)	1.6 (+/− 0.7)	1.0 (+/− 0.3)	3.00	0.005
Total cholesterol (mmol/L)	5.3 (+/− 0.8)	4.6 (+/− 0.6)	4.23	4.48 × 10^−4^
HDL (mmol/L)	1.4 (+/− 0.4)	1.8 (+/− 0.9)	−1.46	0.083
LDL (mmol/L)	3.2 (+/− 1.1)	2.6 (+/− 0.6)	2.71	0.009
Systolic blood pressure (mmHg)	135 (+/− 18)	115 (+/− 9)	3.37	0.003
Diastolic blood pressure (mmHg)	78 (+/− 13)	73 (+/− 9)	1.67	0.061
Time (months)		17.6 (+/− 6.9)		

Data are expressed as means +/− standard deviation, based on a paired t-test and one-tailed *P* value (given that gastric bypass is known to result in a marked reduction in weight and metabolic risk).

### Differential methylation of individual CpG loci

**Figure 2:**
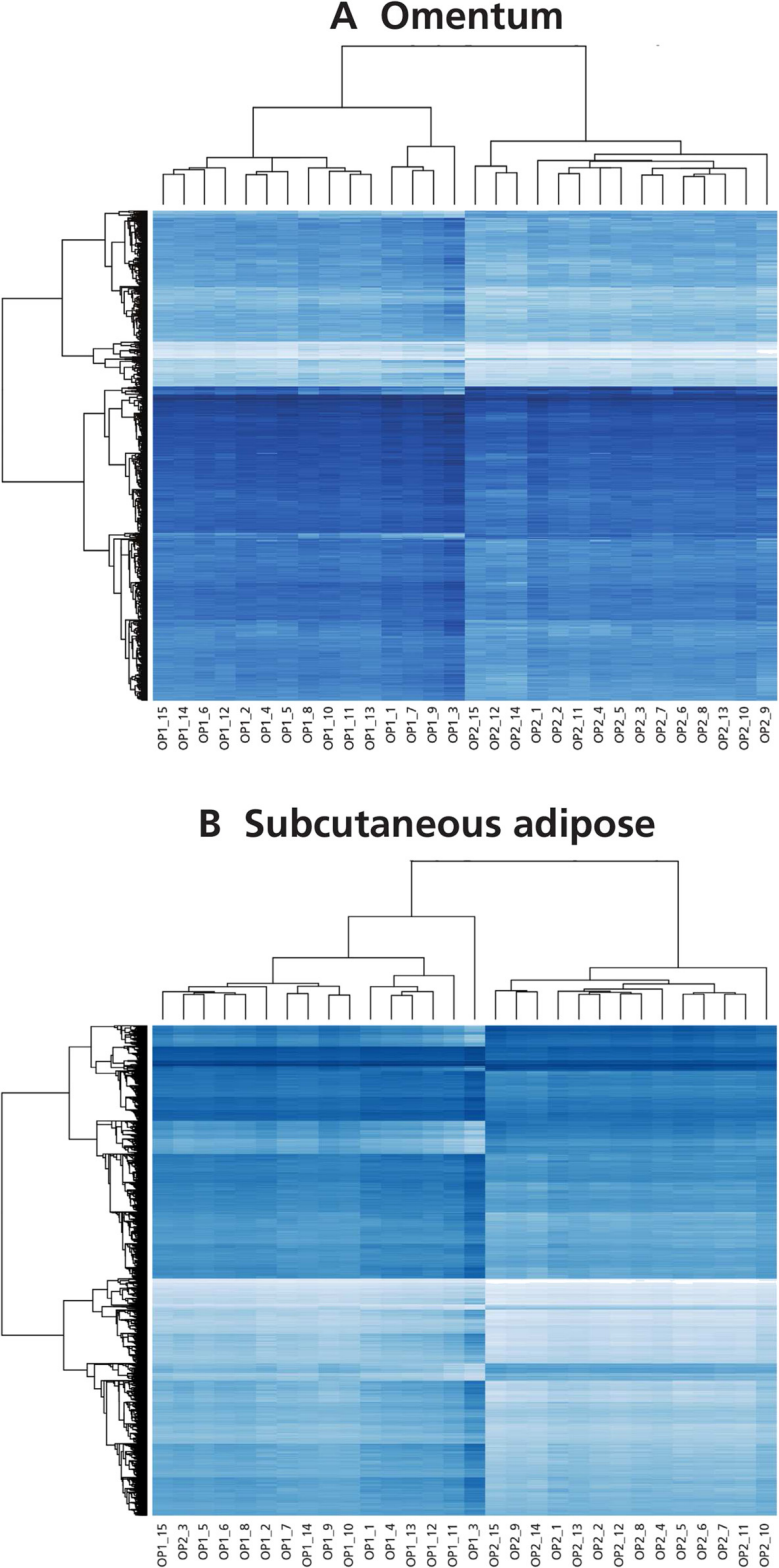
**Heatmap of hierarchical clustering for differentially methylated CpG sites in omentum (A) and subcutaneous adipose (B).** CpG sites passing a relaxed (*P* <1 × 10^−5^) are represented. Dark blue to white indicates hypermethylation through hypomethylation. OP1 and OP2 indicate samples taken before and after gastric bypass, respectively; numeral indicates samples from individuals 1 to 15.

We compared the methylation (beta value) of individual CpG sites before and after weight loss. Initial analysis using a relaxed multiple testing threshold (*P* < 1 × 10^−5^) to interrogate the data revealed 1,347 and 25,729 differentially methylated loci for omentum and subcutaneous adipose, respectively. Unsupervised hierarchical clustering grouped the samples principally by sampling time (before or after weight loss) (Figure 2). When Bonferroni [47] correction was applied (*P* <1 × 10^−7^) 15 and 3,601 differentially methylated CpG sites were observed in omentum and subcutaneous adipose (Additional file 2), respectively.

**Figure 3:**
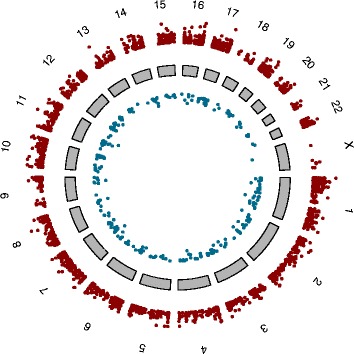
**Genome-wide differential methylation in subcutaneous adipose.** Difference in methylation (∆beta) for all CpG sites passing Bonferroni correction is plotted. ∆beta is weighted by t-statistic such that distance from central core (grey) indicates increasing levels of statistical significance. Red dots represent CpG sites hypermethylated and blue dots CpG sites hypomethylated, respectively, before weight loss. Chromosomes are shown clockwise from 1 through X.

Differentially methylated CpG sites were present across the genome with all chromosomes represented. Figure 3 presents a genome-wide differential methylation plot (∆beta) for CpG sites passing Bonferroni correction in subcutaneous adipose and Table 2 provides summary statistics for both tissues. The top 20 most hyper and hypo methylated CpG sites in subcutaneous adipose mapping to known genes are shown in Table 3 (full dataset, Additional file 2), and the 15 differentially methylated CpG sites passing Bonferroni correction in omentum are presented in Table 4.

**Table 2:** **Summary of differentially methylated CpG sites which passed Bonferroni correction**

**Tissue**	**Number of CpG sites**	**CpG sites mapping to known genes (unique)**	**CpG sites mapping to intergenic regions**	**More methylated before weight loss**	**More methylated after weight loss**	**Absolute range ∆beta**	**Number of CpG sites with ∆beta > 10% (20%)**
Omentum	15	11 (11)	4	14	1	0.034-0.112	1 (0)
Subcutaneous adipose	3,601	2,458 (1,889)	1,143	3,281	320	0.020-0.273	2,401 (145)

**Table 3:** **Top20 CpG sites in known genes which were most hypermethylated or hypomethylated in subcutaneous adipose**

**Ilumina probe ID**	**Gene symbol**	**Chromosome and map position (build 37)**	**∆beta**	**T statistic**	***P*** **value**
cg10838410	*IFFO1*	12: 6659524	−0.223	−10.2	6.8 × 10^−8^
cg27631256	*TNFSF8*	9: 117692745	−0.203	−11.6	1.5 × 10^−8^
cg21011616	*HOXD4*	2: 177015992	−0.188	−10.9	3.0 × 10^−8^
cg22491058	*C4BPA*	1: 207277466	−0.182	−11.4	1.8 × 10^−8^
cg07787634	*KMO*	1: 241715378	−0.177	−10.3	6.7 × 10^−8^
cg07420362	*TRPC2*	11: 3647419	−0.176	−10.5	5.1 × 10^−8^
cg14012082	*EPX*	17: 56274407	−0.176	−11.9	1.0 × 10^−8^
cg17518550	*C1orf150*	1: 247712383	−0.175	−10.3	6.3 × 10^−8^
cg13053653	*HOXD3*	2: 177037631	−0.175	−11.0	2.8 × 10^−8^
cg08400424	*ARHGAP26*	5: 142357042	−0.172	−10.2	7.7 × 10^−8^
cg11236746	*GLB1*	3:33096255	−0.172	−10.5	5.4 × 10^−8^
cg09359351	*HSPBAP1*	3:122509111	−0.171	−10.1	8.1 × 10^−8^
cg14118850	*HPCAL1*	2:10447890	−0.171	−14.5	7.9 × 10^−10^
cg20426866	*TACC2*	10:123923881	−0.170	−12.1	8.6 × 10^−9^
cg08055663	*KCTD5*	16:2737356	−0.169	−10.0	9.5 × 10^−8^
cg08949974	*RBM47*	4:40632860	−0.167	−10.0	9.0 × 10^−8^
cg02794695	*SLA; TG*	8:134072611	−0.166	−10.0	9.2 × 10^−8^
cg11967765	*GNA12*	7:2774195	−0.165	−10.0	9.7 × 10^−8^
cg13453168	*PTPRE*	10:129845660	−0.165	−11.8	1.2 × 10^−8^
cg26933869	*ALDH4A1*	1:1921807	−0.164	−10.0	9.3 × 10^−8^
cg10111816	*CDR2*	16: 22384715	0.273	12.1	8.3 × 10^−9^
cg07852840	*SPSB4*	3: 140813919	0.273	11.0	2.8 × 10^−8^
cg17219660	*GPR37L1*	1: 202091880	0.269	10.7	3.9 × 10^−8^
cg08344351	*CMIP*	16: 81507328	0.269	10.0	8.8 × 10^−8^
cg23753807	*ABR*	17: 1090291	0.265	13.7	1.7 × 10^−9^
cg04546573	*GULP1*	2: 1090291	0.263	10.4	5.8 × 10^−8^
cg08264805	*RUSC1, C1orf104*	1: 155291224	0.254	13.8	1.5 × 10^−9^
cg17691545	*SLC10A6*	4: 87770586	0.254	11.6	1.4 × 19^−8^
cg00838040	*ATP2C2*	16: 84446919	0.244	11.3	2.1 × 10^−8^
cg13052101	*MTL5*	11: 68517529	0.239	11.4	1.7 × 10^−8^
cg10249224	*TSSK3; LOC100128071*	1: 32828191	0.238	11.3	2.1 × 10^−8^
cg09869811	*NUTF2*	16:67897346	0.237	13.4	2.3 × 10^−9^
cg14563485	*ZFP14*	19:36860285	0.236	11.5	1.7 × 10^−8^
cg02363593	*DOCK2*	5:169123192	0.236	11.8	1.2 × 10^−8^
cg18573082	*PDE7B*	6:136425179	0.236	12.4	6.0 × 10^−9^
cg15174117	*CORO2B*	15:68913662	0.235	11.8	1.1 × 10^−8^
cg05939149	*PLEKHH2*	2:43986106	0.235	10.6	4.4 × 10^−8^
cg27083087	*VGLL4*	3:11610239	0.234	10.6	4.4 × 10^−8^
cg07080653	*LNX1*	4:54374249	0.233	11.7	1.2 × 10^−8^
cg17080882	*TGFBR3*	1:82191625	0.232	10.6	4.8 × 10^−8^

A positive value for ∆beta indicates hypermethylation before weight loss in comparison to after weight loss, and a negative value indicates hypomethylation.

**Table 4:** **Differentially methylated CpG sites in omentum**

**Ilumina probe ID**	**Gene symbol**	**Chromosome and map position (build 37)**	**∆beta**	**T statistic**	***P*** **value**
cg04627183	*PDE7B*	6: 136365249	0.070	13.5	2.0 × 10^−9^
cg08351331	*LBP*	20: 36975083	0.058	12.1	8.5 × 10^−9^
cg08514779	-	13: 112076555	0.073	11.9	1.1 × 10^−8^
cg16546882	-	7: 54731606	0.037	10.8	3.7 × 10^−8^
cg01907005	*PLIN4*	19: 4517804	0.043	10.5	5.0 × 10^−8^
cg00031105	*RXRB*	6: 33166117	0.069	10.5	5.2 × 10^−8^
cg06673130	*CPLX1*	4:778924	−0.072	−10.5	5.2 × 10^−8^
cg18736186	*GALK2*	15: 49462118	0.054	10.4	5.6 × 10^−8^
cg19758873	-	4: 49462118	0.059	10.2	7.6 × 10^−8^
cg06579248	*MYO1C*	17: 1375968	0.034	10.1	8.0 × 10^−8^
cg26790198	*CSTL1*	20: 23419120	0.041	10.1	8.7 × 10^−8^
cg15420634	*SLC9A3*	5: 485359	0.041	10.1	8.8 × 10^−8^
cg01127300	-	22: 38614796	0.112	10.0	9.3 × 10^−8^
cg04344695	*CA12*	15: 63658391	0.091	10.0	9.7 × 10^−8^
cg12157387	*PARD3B*	2: 206347777	0.083	10.0	9.7 × 10^−8^

All CpG sites passing Bonferroni correction are shown. A positive value for ∆beta indicates hypermethylation before weight loss in comparison to after weight loss, and a negative value indicates hypomethylation. Gene symbol ‘-’ represents an intergenic region to which no known genes map.

When we looked for an overlap between the 15 and 3,601 robustly identified CpG sites in omentum and subcutaneous adipose, respectively, we identified one (Illumina probe cg08514779), located in an intergenic region on chromosome 13 (∆beta 0.12 subcutaneous adipose, ∆beta 0.07 omentum). Two other genes, *PDE7B* and *SLC9A3*, had differentially methylated sites in both tissues but not at the same CpG loci.

Next we looked at the number of genes which had multiple differentially methylated CpG sites. Of the 3,601 CpG sites in subcutaneous adipose (corresponding to 1,889 unique genes) 438 genes had >1 and 15 genes had ≥6. Of these 12/15 showed consistent hypermethylation of all CpG sites before weight loss (*GNG7* 9 CpGs ∆beta 0.04-0.20, *C7orf50* 8 CpGs ∆beta 0.08-0.19, *PRDM16* 8 CpGs ∆beta 0.07-0.16, *TRIM2* 7 CpGs ∆beta 0.12-0.19, *COL11A2* 7 CpG sites ∆beta 0.07-0.15, *FOXP2* 6 CpGs ∆beta 0.09-0.20, *FRMD4A*, 6 CpG sites ∆beta 0.10-0.17, *GPR37L1* 6 CpGs ∆beta 0.14-0.27, *KCNQ1* 6 CpGs ∆beta 0.06-0.10, *RASA3* 6 CpGs ∆beta 0.07-0.15, *THBS1* 6 CpGs ∆beta 0.12-0.20, *TNXB* 6 CpGs ∆beta 0.05-0.15). Three genes showed hypomethylation of one CpG before weight loss and hypermethylation of the remaining CpGs (*PRKCZ* 8/9 CpG ∆beta 0.08-0.16, 1/9 ∆beta −0.10, *PTPRN2* 7/8 CpGs ∆beta 0.07-0.17, 1/8 ∆beta −0.11, *SHANK2* 5/6 CpG ∆beta 0.9-0.10, 1/6 ∆beta −0.16*).* Further information regarding these sites can be found highlighted within Additional file 2.

To further investigate genomic regions with consistent, extended differential methylation we used the probe lasso approach as implemented in ChAMP [48]. This analysis was performed on probes passing a more relaxed adjusted *P* value, Benjamini-Hochberg rather than Bonferroni, in order to increase the number of sites for DMR (differentially methylated region) identification. Analysis of subcutaneous adipose revealed 195 DMRs passing a nominal *P* <0.05. These DMRs mapped to 162 annotated loci (Additional file 3), including five with two DMRs (*TNXB*, *PRKCZ*, *IQCE*, *LRRC17/FBXL13*, *HOXC4*). Of the 15 genes in which we observed ≥6 probes passing Bonferroni correction DMRs were identified in eight, which reflects the method of DMR detection implemented in the ChAMP probe lasso protocol (in particular the size of the lasso and *P* value of the individual sites). Equivalent probe lasso analysis of the omental adipose data did not reveal any DMRs.

**Figure 4:**
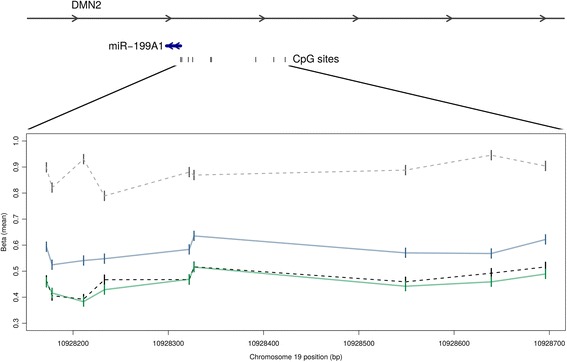
**An example DMR upstream of miR-199A1.** Location and orientation of miR-199A1 within *DMN2*, as well as CpG sites upstream of the miRNA are shown above. The bottom panel shows average beta values across the DMR on chromosome 19 for: healthy whole blood (light grey dotted), subcutaneous adipose before weight loss (blue), subcutaneous adipose after weight loss (green), and healthy subcutaneous adipose (black dotted).

Given that adipose tissue is by its nature a mixture of cells, and that inflammation is known to play an important role in obesity, we hypothesised that some differentially methylated loci might correspond to epigenetic signatures within inflammatory cells. We therefore compared the DMRs in our subcutaneous tissue samples, with the same regions in publicly available whole blood (as a proxy for inflammatory cells) and publicly available healthy subcutaneous adipose tissue. Figure 4 presents data for a DMR where the methylation profile of the preoperative (more obese) tissue is higher than those for postoperative and healthy tissue, and is thus potentially ‘pulled’ towards the whole blood profile by its complement of inflammatory cells.

#### Differential methylation of genes associated with obesity, type-2 diabetes and related traits

To investigate the potential biological significance of our findings we interrogated the genes to which differentially methylated CpGs mapped against the catalogue of published Genome-wide association studies (GWAS) [49], and identified loci associated with obesity and related metabolic traits for 120 genes in subcutaneous adipose (Table 5). We also identified six DMRs which mapped to obesity and related metabolic traits in the GWAS catalogue (*PROX1*, *PHACTR1*, *SLC22A8*, *SMAD3*, *LCAT*, *DNM2*). Further refining our search we investigated obesity and type-2 diabetes candidates listed by McCarthy [15]. Of 57 obesity and 60 type-2 diabetes loci all but one were present on the Illumina 450 K platform. We saw differential methylation within 9/57 obesity (single CpG sites within: *LEPR*, *STAB1*, *ZNF608*, *HMGA1*, *MSRA*, *TUB*, *NRXN3*, *FTO*, *MC4R*) and 10/59 type-2 diabetes (single CpG sites: *PROX1*, *RBMS1*, *IRS1*, *TCF7L2*, *FTO*, *LMNB2*, *INSR*. Two CpG sites within *AKT2*, *BCL11A.* Six CpG sites within *KCNQ1*) genes in subcutaneous adipose. A DMR was identified in one of the type-2 diabetes genes (*PROX1)* and differential methylation of the six CpG sites in *KCNQ1* were validated by pyrosequencing (see later).

**Table 5:** **Overlap between differentially methylated loci in subcutaneous adipose and genes associated with obesity and related traits**

**Obesity-related trait**	**GWAS associated genes with at least one DM CpG site**	**GWAS specific trait(s)**	**PMID (PubMed IDs)**
BMI and weight	*RASAL2*; *ADCY3*; *KCNE4*; *DGKG*; *ZNF608*; *HMGA1*; *AIF1*; *TUB*; *NRXN3*; *ADCY9*; *FTO*; *SEPT9*; *MC4R*; *FOXN3*	Body mass index, Weight; Weight loss (gastric bypass surgery)	17434869, 18454148, 19079260, 19079261, 20935630, 20966902, 22344219, 22344221, 22982992; 23643386; 23583978, 23563607
Waist circumference	*KCNE4*; *PPM1H*; *NRXN3*; *FTO*; *MC4R*	Waist circumference	19557197, 20966902
Metabolic syndrome	*GALNT2*; *MICB*; *CAMK2B*; *TMEM195*; *TCF7L2*; *STOML3*; *LIPC*; *PKMYT1*; *FTO*; *CETP*	Metabolic syndrome, Metabolic syndrome (bivariate traits)	20694148, 21386085, 22399527
Metabolic traits	*LEPR*; *ENPEP*	Metabolic traits	19060910, 21886157
Blood pressure	*CASZ1*; *CLCN6*; *CNTN4*; *MECOM*; *ENPEP*; *EBF1*; *BAT2*; *MSRA*; *CACNB2*; *C10orf107*; *PLCE1*; *PLEKHA7*; *FLJ32810*; *ATP2B1*; *PTPN11*; *ACBD4*; *PLCD3*; *MC4R*	Blood pressure, Obesity and blood pressure, Diastolic blood pressure, Systolic blood pressure, Hypertension	17903302, 19430479, 19430483, 21572416, 21909110, 21909115, 22013104
Cardiovascular	*KLHL29*; *TCF7L2*; *CADM1*; *APOA4*; *ATP2B1*; *C12orf51*; *COL4A1*; *LIPC*; *SMAD3*; *CETP*; *LCAT*; *ZFHX3*; *SMG6*; *UBE2Z*; *DNM2*; *STX16*; *MRPS6*; *FLT1*; *CUX2*; *PTPN11*	Cardiovascular disease risk factors, coronary heart disease	17634449, 20838585, 21347282, 21378990, 21943158, 22751097, 23364394
Type-2 diabetes	*BCL11A*; *RBMS1*; *IRS1*, *MAEA*; *AKAP2*; *CAMK1D*; *TCF7L2*; *GRK5*; *TCERG1L*; *KCNQ1*; *FTO*; *CMIP*; *PALM2*; *TGFBR3*; *WISP1*; *SND1*	Type-2 diabetes, Type 2 diabetes and other traits	17293876, 17460697, 17463246, 17463248, 17463249, 17554600, 17668382, 18372903, 18711366, 18711367, 19056611, 19404141, 19734900, 20174558, 20418489, 20581827, 20862305, 20864672; 21490949, 21573907, 21799836, 22101970, 22158537, 22456796, 22693455, 22931080, 22961080, 23209189, 23300278; 23532257
Blood lipids	*MACF1*; *GALNT2*; *IRS1*; *AFF1*; *ARL15*; *NPC1L1*; *DNAH11*; *PINX1*; *XKR6*; *APOA1*; *APOA4*; *MYO1H*; *C12orf51*; *LIPC*; *CETP*; *LCAT*; *CMIP*; *MC4R*; *NCAN*	Cholesterol, Cholesterol total, HDL cholesterol, LDL cholesterol, Triglycerides	17463246, 18193043, 18193044, 19060906, 19060910, 19060911, 19074352, 19359809, 20031538, 20031564, 20066028, 20686565, 20864672, 21909109, 23505323, 20139978
Adipose tissue	*TP73*; *KIAA0495*; *ITPKB*; *IRS1*; *LPP*; *ZNF608*; *CNTNAP2*; *MSRA*; *BICD2*; *PRICKLE1*; *RORA*; *SMAD6*; *FTO*	Adiposity, Visceral adipose tissue/subcutaneous adipose tissue ratio, Subcutaneous adipose tissue, Visceral fat	19557161, 21706003, 22589738
Blood glucose	*PROX1*; *RREB1*; *TMEM195*; *TCF7L2*; *IGF1*, *FOXN3*	Fasting glucose-related traits (interaction with BMI), Fasting glucose-related traits	20081858, 22581228
Insulin	*IRS1*; *SLC10A6*; *TCF7L2*; *TCERG1L*; *IGF1*; *ATP10A*; *LARP6*; *PCBP3*	Fasting insulin-related traits (interaction with BMI), Insulin-related traits, Fasting insulin-related traits, Proinsulin levels, Insulin resistance/response	20081858, 21873549, 21901158, 22581228, 22791750

Genes were identified as associated with obesity and related traits in the GWAS catalogue [49].

When we investigated the list of 162 DMRs mapping to annotated loci (Additional file 3) in further detail we observed other genes with known and potential roles in obesity and related traits (such as *PRKCZ*, *NDUFS2*, *GATA2*, *FOXP2*, *ANGPT2*, *NCOR2*, *CPT1B*, *PTPN6*). Notably DMRs were also observed in two brown adipose related genes *PRDM16* and *Acot11*. In addition, we observed DMRs encompassing loci for miR429, miR657, miR338 and miR199A and antisense RNA locus, *EMX20S* (Additional file 3).

None of the 11 genes showing robust differential methylation in omentum mapped to entries in the GWAS catalogue or candidate loci listed by McCarthy [15]. However, 4/11 have known or potential roles in obesity, *MYO1C*, *PLIN4*, *PARD3* and *PDE7B* [50-62].

### Differential methylation of genes involved in epigenome regulation

DNA methylation and histone modification are two epigenetic mechanisms and we observed differential methylation of a number of genes involved in these processes. DNA methyl-transferases actively methylate cytosines in CpG dinucleotides. In subcutaneous adipose we saw differential methylation of two CpG sites within DNA methyl-transferase *DNMT3A* (∆beta 0.11, 0.08) and *DNMT3L* (∆beta 0.09) which can act with *DNMT3A* to stimulate *de novo* methylation, and also interacts with histone deacetylase 1. Furthermore we observed differential methylation of one CpG in *MBD4* (∆beta 0.08), a methyl-CpG-binding domain protein and DNA glycosylase with excision activity against 5-methyl cytosine. In addition, single CpG loci within *HDAC7* (∆beta 0.08) and *HDAC10* (∆beta 0.06) were more methylated before weight loss, with *HDAC4* showing a mixed differential methylation signature, two CpG sites hypermethylated (∆beta 0.09, 0.10) and one hypomethylated (∆beta −0.11) before weight loss. Differential methylation was not observed for any genes encoding other histone modification enzymes.

None of the 11 genes with robust differential methylation in omentum encode known DNA methyl-transferases, DNA demethylation enzymes or histone modification enzymes.

### Differential methylation of homeobox genes

Metabolic risk is affected by both the distribution of body fat between different adipose tissue depots within an individual and the differing natures of the depots themselves [9-11]. Homeobox containing genes play a key role in the development and function of tissues, and one class of homeobox genes, *HOX*, in particular have been shown to be expressed in adipose tissue in a depot specific manner [63]. Therefore, we investigated differential methylation of CpG sites within *HOX*, and also *PAX*, *MSX* and *EMX* homeobox-containing genes.

In subcutaneous adipose differential methylation of multiple CpG sites within 3/4 *HOX* gene clusters was observed: *HOXA4* (three CpG sites ∆beta 0.09, 0.07, 0.07) and *HOXA3* (two CpG sites ∆beta 0.21, 0.12) on chromosome 7; *HOXB1* (∆beta 0.11), *HOXB3* (∆beta 0.12) and *HOXB6* (two CpG sites ∆beta 0.13, 0.11) on chromosome 17; and *HOXD4* (four CpG sites ∆beta −0.13, −0.14, −0.19, −0.14) and *HOXD3* (∆beta −0.18) on chromosome 2. Differential methylation was also seen for single CpG sites in *MSX1* (∆beta −0.15) and anti-sense transcript *EMX20S* (∆beta −0.12) which overlaps *EMX2*. Furthermore, we also observed DMRs showing hypermethylation before weight loss within *HOXA3* (Chr7:27153534–27153784, 5′UTR), *HOXC4* (Chr12: 54409141–5447404, TSS1500, and Chr12: 5447099–5447404, 5′UTR) and a larger DMR in *HOXB6* (Chr17:46681413–46683953, gene body). A DMR showing hypomethylation before weight loss was identified in *HOXD3* (Chr2:177030029–177030349, 5′UTR) (Additional file 3).

No differential methylation of homeobox containing genes was observed for robust differentially methylated CpG sites in omentum. When a relaxed *P* value was used (*P* <1 × 10^−5^) only one site in *HOXA3* (∆beta 0.08, *P* = 7.3 × 10^−6^) and another in *MSX1* (∆beta −0.13, *P* = 7 × 10^−6^) were seen.

### Pathways enrichment analysis for differentially methylated genes

To investigate the potential biological relevance of the differentially methylated CpGs we performed initial enrichment analyses of all loci. For subcutaneous adipose, analyses were carried out on the 1,889 genes to which the 3,601 CpG sites passing Bonferroni correction mapped. Enrichment was seen in Gene Ontologies (GO): phospholipid binding (GO:0005543, 96 genes, *P* = 4.1 × 10^−10^); GTPase regulator activity (GO:0005520, 87 genes, *P* = 5.9 × 10^−9^); actin binding (GO: 0003779, 70 genes, *P* = 3.9 × 10^−7^); Ras protein signal transduction (GO:0007265, 74 genes, *P* = 1.1 × 10^−6^), and insulin-like growth factor binding (GO:0019838, 10 genes, *P* = 0.01). Further pathways showing enrichment included: focal adhesion (36 genes, 1.9 × 10^−13^); metabolic pathways (99 genes, *P* = 2.7 × 10^−12^); TGFβ signalling (13 genes, *P* = 6 × 10^−6^); insulin signalling (21 genes, *P* = 8.3 × 10^−5^); leptin signalling (12 genes, *P* = 0.0001); and adipogenesis (14 genes *P* = 0.026). All *P* values are Bonferroni adjusted.

We also investigated whether any of the groups of CpG sites identified by the hierarchical clustering (Figure 2) showed enrichment. Only clusters containing >70 genes were included (7/13) and enrichment for glucoronidation, insulin and EGF/EGFR signalling, TGFβ and AMPK signalling, lymphocyte and T cell apoptosis, cell signalling and plasma membrane was observed.

Given that DNA methylation and DNA demethylation are regulated by different mechanisms we also separated the loci according to whether they were relatively hyper- or hypomethylated before gastric bypass and weight loss. GO analysis revealed that hypomethylated loci were enriched for immune response-related function (for example, GO: 0006955, *P* = 0.0001) which was not seen for the hypermethylated genes; these showed enrichment for phospholipid binding, actin binding and GTPase regulator activity as above.

Due to the small number of CpG sites passing Bonferroni correction in omentum we used the more relaxed threshold (*P* <1 × 10^−5^) for enrichment analyses (920 unique genes to which 1,347 CpG sites mapped). GO enriched were: GTPase regulator activity (GO: 0030695, 53 genes, *P* = 1.6 × 10^−8^); actin cytoskeleton (GO: 0015629, 33 genes, *P* = 0.009) and phospholipid binding (GO: 0005543, 41 genes, *P* = 0.015). Enriched pathways included focal adhesion (15 genes, *P* = 2.5 × 10^−5^) and insulin signalling (10 genes, *P* = 0.008). All *P* values are Bonferroni adjusted.

Hierarchical clustering of the CpG sites passing the relaxed threshold in omentum revealed 15 clusters, five of which had >70 genes. Enrichment for GTPase regulator activity, G-protein signalling and TGFβ and insulin signalling was observed.

Separation of the loci into those hyper and hypo methylated before gastric bypass revealed enrichment for 2/60 hypo-methylated loci (*SORT1* and *PCSK6*, related to nerve growth and neutrophin binding, GO: 0048406 and GO: 0043121, respectively). The hypermethylated loci were enriched for GTPase regulator and related activities also seen in the subcutaneous adipose analysis.

### Environmental factors involved in differential methylation of adipose tissue

Clinical blood measures (such as elevated fasting glucose, insulin and lipids) are not only indicators of disease risk but also provide an indication of the internal body environment. Therefore, potential relationships between changes in methylation and changes in 11 clinical variables (weight, BMI, fasting glucose, insulin, blood lipids (HDL, LDL, triglycerides, total cholesterol), HbA1c, and systolic and diastolic blood pressure) were investigated using Pearson’s correlation. In order to maximise the likelihood of identifying robust correlations we filtered our analysis at absolute R >0.75 and *P* <0.05 (Benjamini Hochberg [64]). Of the 1,889 annotated loci to which the 3,601 CpG sites passing Bonferroni correction mapped we observed correlations between methylation in 40 genes and at least one clinical trait (Additional file 4). Correlations between genes and weight loss were observed in two genes with known roles in obesity: *FOXP2* (cg18546840 R = 0.85) and *ACSL1* (cg00287477 R = 0.91). A correlation between change in methylation and weight was also seen for *CELSR1* (cg06652313, R = 0.82). In addition, methylation changes at a number of loci which did not show a strong correlation with weight loss did show strong, independent correlations with other clinical traits such as fasting glucose (Additional file 4). With ∆fasting glucose we observed correlations with change in DNA methylation for *HDAC4* (cg26078407 R = −0.75) and *SLC37A3* (cg15751131 R = −0.80). In addition, a correlation between *DENND1C* (cg11599981) and both fasting glucose and HbA1c (R = 0.75 and 0.85, respectively) was seen.

An interrogation of the 15 CpG sites from omentum did not reveal any correlations passing stringent filtering, or at a more relaxed R >0.5.

### Technical validation and extension of DNA methylation analyses

**Figure 5:**
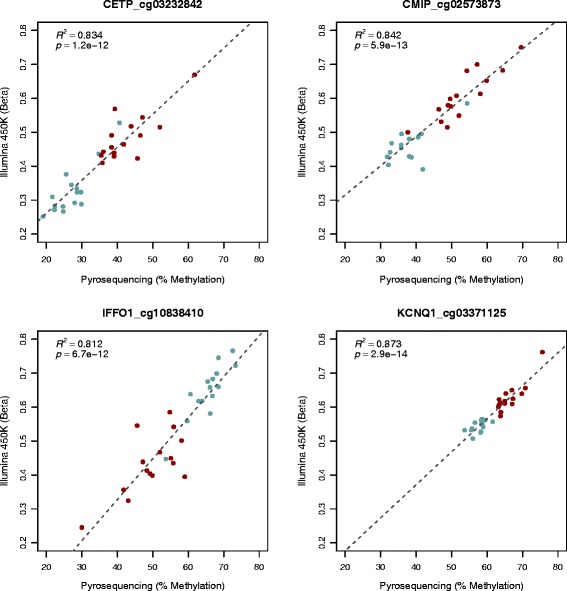
**Correlations between Illumina 450 K array data and pyrosequence analysis.** Representative data for a single CpG site in four genes are shown. Red indicates samples taken before and blue after gastric bypass. Illumina probe IDs are indicated after gene names.

We analysed a number of loci using an independent DNA methylation assay, pyrosequencing, in the 15 individuals from the array experiment. Six genes/loci were selected from the subcutaneous adipose analysis based on a number of criteria: (1) most hyper- and hypomethylated before and after weight loss (*CMIP* and *IFFO1*, see Table 3); (2) genes implicated in obesity and/or type-2 diabetes (*KCNQ1*, *CETP*, *CMIP*, *ADRAB2*); and/or (3) reported differential adipose mRNA expression in response to weight loss/dietary intervention *(CETP*, *CTGF*). All CpG probe sites passing Bonferroni correction in a given gene were analysed. Correlation between the two types of methylation assay for all 15 sites interrogated was good (R^2^ = 0.656-0.939, *P* = 3.6 × 10^−8^ - 9.8 × 10^-19,^ Additional file 5). Figure 5 presents data for four representative CpG loci.

For omentum we selected the CpG probe sites in the four genes with known or potential roles in obesity *MYO1C*, *PLIN4*, *PARD3* and *PDE7B*. Significant correlations between methylation measures was observed for *PDE7B* (R^2^ = 0.671, *P* = 1.9 × 10^−8^), *PLIN4* (R^2^ = 0.399, *P* = 0.0001) and *PARD3B* (R^2^ = 0.528, *P* = 2.4 × 10^−6^). Differential methylation of *MYO1C* did not validate (R^2^ = −0.02, *P* = 0.49) although the original assay failed and the second design did not seem as robust as other assays.

DNA was also available from samples taken before and after weight loss for an additional 12 individuals for subcutaneous adipose (9 men, 3 women) and an additional seven for omentum (5 men, 2 women). A combined analysis of the original samples and extended cohort revealed significant differential methylation of all 15 CpG sites interrogated in subcutaneous adipose in women and men except two CpGs in *KCNQ1* which were not differentially methylated in men (Additional file 6). A comparison of methylation at each CpG between men and women revealed small but statistically significant differences (*P* <0.05) at a number of loci (Additional file 6). Notably, six CpGs within an island in *CMIP* all showed statistically more methylation in women than men after weight loss as did three adjacent CpG sites in *CETP.*

In omentum the CpG loci within *PARD3B*, *PDE7B* and *PLIN4* showed differential methylation before and after weight loss in men and women except one CpG site located 7 bases upstream of the transcription start site of *PDE7B* (Additional file 7). None of the CpG loci within *PARD3B*, *PDE7B* and *PLIN4* showed a significant difference in methylation level between men and women (Additional file 7).

### mRNA analysis of differentially methylated genes

DNA methylation is known to affect transcription [65]. Therefore we wanted to examine mRNA expression of genes to which the differentially methylated CpG sites mapped. Because of the number of CpG sites passing multiple testing adjustment, we chose to limit our analysis to those located in gene promoter regions. Defining promoter regions broadly as the 5′UTR, or within 1,500 bases upstream of the transcription start site (5′UTR, TSS200 and TSS1500 as per Illumina annotation) we identified 777/1,889 genes in subcutaneous adipose with differential methylation in their promoter regions. We selected five of these (*ACACA*, *CETP*, *CTGF*, *S100A8*, *S100A9*) for further analyses based on reported adipose or adipocyte gene expression changes in response to weight loss/dietary intervention (*ACACA*, *CETP*, *CTGF* [66-69]), and/or associations with obesity or related traits (GWAS, and/or other literature). *CTGF*, *S100A8* and *S100A9* were upregulated and *CETP* downregulated before weight loss. Expression of *ACACA*, *S100A8*, *S100A9* and *CETP* was negatively correlated with DNA methylation, that is less expression was observed with more methylation, whereas mRNA levels of *CTGF* were positively correlated (Table 6).

**Table 6:** **mRNA expression and DNA methylation in subcutaneous adipose and omentum**

**Gene**	**Adipose tissue**	**log2 fold change expression before vs. after weight loss**	**T statistic**	***P*** **value**	**∆ DNA methylation before vs. after weight loss**	**Methylation probe location**
*ACACA*	Subcutaneous	−0.82	4.02	0.001	−0.17	5′ UTR
*CETP*	Subcutaneous	−2.48	5.49	0.00008	0.15	TSS1500
*CTGF*	Subcutaneous	0.94	3.72	0.002	0.14, 0.12	TSS1500
*S100A8*	Subcutaneous	2.49	4.46	0.0005	−0.12	5′UTR
*S100A9*	Subcutaneous	2.23	4.18	0.0009	−0.12	5′UTR
*PLIN4*	Omentum	0.89	4.86	0.0002	0.04	TSS200

Two of 11 genes identified in omentum had differential methylation within their promoter regions (*PLIN4*, *GALK2*). *PLIN4* was selected for further investigation because it is associated with obesity, insulin resistance and increased blood lipids [55-60,70]. mRNA expression was upregulated before compared to after weight loss, as was methylation (Table 6). As we had pyrosequence for six CpG sites immediately upstream of the *PLIN4* transcription start site we averaged the percent methylation across this region and tested for a correlation (linear regression) with mRNA expression both before and after weight loss. Interestingly we observed a strong correlation after weight loss (R^2^ = 0.55, *P* = 0.0009) with decreased methylation corresponding to increased mRNA expression whereas no correlation was observed before weight loss (R^2^ = −0.03, *P* = 0.5).

## Discussion

This study revealed significant changes in DNA methylation in two distinct abdominal adipose tissue depots before and after gastric bypass and associated weight loss. Global DNA methylation was greater in subcutaneous adipose and omentum before weight loss (Figure 1). This observation is intriguingly in concordance with another comparison before and after gastric bypass, but in skeletal muscle [45], despite the fact that numerous factors could be influencing the global shift such as cell heterogeneity, and environmental changes like metabolic status, lifestyle and diet. It is also possible that this hypomethylation in different tissues after gastric bypass reflects an effect of gastric bypass specifically and/or the profound level of weight loss. This is particularly interesting given the observation that after 6 months exercise intervention in non-obese male individuals an increase in global DNA methylation of thigh subcutaneous adipose was observed [40]. These contrasting observations potentially highlight important differences between adipose tissue depots, in terms of gender, location and context (for example, abdominal vs. thigh, near or distant from greatest reduction in adipose tissue mass, non-obese vs. obese), cellular complexity and functional response to weight loss and/or exercise. Furthermore, DNA demethylation (as evidence by relative hypomethylation) can occur by both active and passive mechanisms [71]. In proliferating cells passive loss of methylation can result from successive rounds of replication in the absence of restoration of CpG methylation by DNMT1 UHFR1, or an initial active oxidation of methylated cytosine followed by replication-dependent dilution [71]. We did observe decreased methylation after gastric bypass and weight loss within one DNA methyl-transferase *DNMT3A*, and its co-factor *DNMT3L* as well as within the gene for *MBD4* a methyl-cytosine binding protein and DNA glycosylase. Unfortunately, an assessment of DNA replication in our tissue samples was not possible, but the relative contribution replication rate differences may play both in the tissues before and after weight loss and in the different cell types warrants further investigation. Such studies will be particularly important given the additional, recent observation that changes in the metabolic state of cells may affect the levels of enzymatic co-factors involved in active DNA demethylation [72].

The tissues we have examined are by their very nature of mixed cell composition, and this is integral to their function [73]. Adipose tissue contains many different cell types including adipocytes that make up about 20% to 40% of the total cellular content, and fibroblasts, preadipocytes, stem cells and immune cells. This cell milieu will change significantly on weight loss. As evidenced by the pathways enrichment and DMR analyses (Figure 4) a proportion of the observed DNA methylation changes are likely to reflect these changes in cellular composition. While some of these cell-proportion related changes might be seen as bystander effects it is important to recognise that others may highlight important biological and functional differences related to disease. Furthermore, analysis of epigenetic marks, such as DNA methylation, which play a key role in cell development and differentiation, may have increased utility to uncover previously undetected cell-type specific differences of relevance to biology. In addition, we observed differential methylation of a significant number of obesity related genes (not only related to tissue remodelling, or inflammation) which we believe supports our approach.

Future analyses which focus on particular cell types, such as adipocytes and adipose tissue macrophages, and their relative contribution to disease will no doubt yield additional insights. As it is not always possible to access human tissue samples with sufficient speed or of sufficient quantity to isolate specific cell types, algorithms currently being developed by a number of groups to deconvolute cell mixtures in DNA methylation analysis [74-76] will hopefully provide additional insights from whole tissue samples.

Our analysis revealed distinct differences in the level of differential methylation observed for subcutaneous adipose and omentum. One hundred and ninety-five extended DMRs, and robust differential methylation of 3,601 CpG sites were identified in subcutaneous adipose, in comparison to omentum where no DMRs and 15 differentially methylation CpGs were observed. Subcutaneous abdominal adipose can expand significantly to store excess lipid and act as a potential buffer protecting other organs and adipose tissue depots from lipid accumulation [7,8]. Thus, the magnitude in terms of both number of differentially methylated loci and degree of difference (up to 27.4% (Table 2)) we observed potentially reflects the function (and relative plasticity) of this tissue and the large reduction in adipose tissue mass and inflammatory cells, as well as tissue remodelling, that would occur with profound weight loss; a hypothesis supported by the pathways analyses. In addition, the pathways analysis highlighted differences between relatively hyper- and hypomethylated loci before weight loss. Genes involved in the immune response were enriched in the hypomethylated group, potentially reflecting a larger proportion of immune cells in this tissue depot before weight loss.

Expansion of omentum is associated with increased risk of metabolic dysfunction [12-14]. Our observation of significantly less differential methylation in omentum may be indicative of the different function of this organ and that it is potentially more tightly controlled with less tolerance for disruption. Therefore, it is interesting that the pathways analysis of differentially methylated loci passing a relatively relaxed *P* value in omentum (*P* = 1 × 10^−5^) and hyper-methylated before weight loss revealed enrichment of GTPase regulator activity as seen for subcutaneous adipose where the more robust cutoff was used. Adipose tissue is an endocrine organ and has the ability to communicate with other cell types and alter the behaviour of cells within its vicinity [73]. The differences between the two tissues studied here is likely to reflect this; one notable difference being that blood supply to the omentum drains directly into the hepatic portal system and is delivered directly to the liver, another highly metabolic organ.

Four of the 11 genes each with a single differentially methylated CpG site in omentum have known or potential roles in obesity. MYO1C is an actin-based motor protein involved in GLUT4 translocation, and insulin-dependent actin filament remodelling [51-53,56,61]; a SNP variant in *PARD3* has been associated with type-2 diabetes [50]; members of the *PDE7B* gene family (*PDE3B* in particular) play a central role in the regulation of lipolysis, lipogenesis and glucose uptake [54,62], and SNP variants in *PLIN4* have been associated with obesity, insulin resistance and increased blood lipids [55,57-60]. This raises the question as to which of the remaining 7/11 genes (*LBP*, *RXRB*, *CPLX1,* GLAK2, *CSTL1*, *SLC9A3*, *CA12*) may also have a role in obesity. Furthermore, given our observation of differential methylation of a single CpG within the *PLIN4* promoter (Table 4) we performed mRNA analysis and observed increased expression before weight loss, consistent, all be it in a different tissue, with the observation that *PLIN4* knock-down in mice resulted in less lipid accumulation in cardiac tissue [77]. DNA methylation within promoter regions is associated with transcriptional repression [65]. A strong correlation in agreement with this was observed between average methylation across 6 CpG sites adjacent to the *PLIN4* transcription start site (pyrosequencing) and mRNA expression in omentum after weight loss whereas no such correlation was seen for pre-gastric bypass tissue. This may reflect a dysregulation of ‘normal’ *PLIN4* control in the obese omentum. To our knowledge this is the first report for a potential role of DNA methylation in the regulation of obesity associated gene *PLIN4*.

Few studies have looked at DNA methylation in omentum. Methylation of nine CpG sites in the gene body of *DPP4* (exon 2: Chr2:162929997–162929799. Map position as genome build 37/hg19 throughout manuscript) in visceral adipose tissue (sampled from the greater omentum) has been associated with increased plasma lipid [78]. DPP4 is thought to play a role in glucose homeostasis and type-2 diabetes by inactivating incretin hormones involved in glucose-dependent insulin secretion [79,80]. We saw hypermethylation of one CpG in the body of *DPP4* (Chr2:162927003, 2,796 bases away from the sites identified above) using a relaxed threshold before weight loss (∆beta 0.106, *P* = 3.7 × 10^−6^). Given that plasma lipid profile and weight decrease after gastric bypass our results are consistent with the previously observed association with plasma lipid [78], highlighting the potential role of DNA methylation in *DPP4* regulation in omentum for further investigation.

A total of 3,601 individual CpG sites showed significant differential methylation in subcutaneous adipose tissue, mapping to 1,889 unique annotated loci as well as intergenic regions. Thus, for the majority of annotated loci we observed differential methylation at a single CpG within the gene. As a stringent statistical threshold was used selection of candidates for future analyses would benefit from an interrogation of the methylation profile of other Illumina probes within the gene of interest at a more relaxed threshold, as well as indications from the literature (such as those discussed below) with respect to previously reported DNA methylation variation and/or a potential role of biological relevance.

In subcutaneous adipose 120/1,889 differentially methylated loci have been associated with obesity or related traits in GWAS (Table 5), and we saw changes in single CpG sites within 9/57 previously defined obesity genes [15]. Furthermore, Rönn *et al.* recently reported differential methylation of a number of obesity candidate loci in thigh subcutaneous adipose after exercise; we observed differential methylation at single CpG sites within four of these: *NRXN3* (∆beta 0.03, Chr14: 79747774), *STAB1* (∆beta 0.12, Chr3: 52553167), *TUB* (∆beta 0.11, Chr11: 8084670), *ZNF608* (∆beta 0.15, Chr5: 124013536). While Rönn *et al.* did not observe a significant change in BMI they did report a decrease in waist circumference and waist/hip ratio after exercise intervention which suggests a reduction in abdominal adipose mass. Assuming in this case that post-exercise is equivalent to after gastric bypass the direction of DNA methylation change is consistent between the studies for *TUB* (∆beta equivalent 0.034. 11:8102717, 18 kb from CpG site identified in our study) with a greater magnitude of change observed after gastric bypass. In contrast the single CpG sites identified as differentially methylated by Rönn *et al.* in *NRXN3*, *STAB1* and *ZNF408* changed in the opposite direction with relative hypermethylation after exercise intervention. The individual CpGs in *NXRN3* and *ZNF608* were at some distance (>50 kb) from those identified in this study, whereas the sites in *STAB1* are located within 118 bases of each other. This highlights the differences between the study designs (as discussed above), the challenges of interpreting the significance (or not) of robustly identified differential methylation of single CpG sites within genes using genome-wide platforms, and the incompletely understood complexity of the role of DNA methylation in transcriptional regulation; as well as the importance of subsequent validation, more in-depth analysis, and combining different types of data and evidence. This said, the identification of individual CpGs can have real utility for highlighting potential genes of interest as illustrated above for *PLIN4* in omentum where our subsequent pyrosequencing revealed differential methylation of a further five CpGs in the promoter region (Additional file 7) and mRNA analyses revealed changes in gene expression.

At least 4/15 genes containing ≥6 differentially methylated CpG sites had a potential role in obesity and/or related traits (*PRKCZ*, *PRDM16*, *FOXP2*, *THBS1* [81-89]). The DMR analysis also identified extended regions of differential methylation in *PRKCZ*, *PRDM16* and *FOXP2. PRDM16* is of interest and potentially worth further investigation given its role in activating the adipogenesis of brown adipose tissue. Relative hypermethylation was observed before gastric bypass and weight loss within a CpG island located in the body of *PRDM16* (Chr1:305660–3056758). Brown adipose catabolises lipids to produce heat and was thought to essentially disappear in the first year after birth. With the recent discovery that adults retain metabolically active brown adipose tissue has come the suggestion that this tissue could provide a promising target with which to combat obesity [89,90]. A DMR was also identified in the gene body of *ACOT11* (Chr1: 55088744–55089810) which is enriched in brown adipose tissue and *ACOT11* knock-out mice are resistant to diet-induced obesity and show increased energy expenditure [91,92].

Genes in the *HOX* networks are expressed in adipose tissue and appear to have a role in metabolic dysfunction [63]. DNA methylation is known to affect transcription [65], with changes often observed within and adjacent to promoter regions. Of the five DMRs identified within *HOX* genes in this study three were located in 5′ UTR regions (*HOXA3*, *HOXC4*, *HOXD3*) and a second DMR was located within 1,500 bases of the TSS of *HOXC4*.

While our study was not designed or aimed at identifying type-2 diabetes specific DNA methylation, obesity is a significant risk factor for this disease, and type-2 diabetes is substantially reduced in patients after gastric bypass [43,44,93,94]. Therefore, we note that we identified differential methylation of single CpG sites within 7/59, two CpG sites in 2/59 (*BCL11A*, *AKT2*), and consistent changes in six CpG sites in 1/59 (*KCNQ1*) type-2 diabetes genes [15]. A DMR was also identified within one of these, *PROX1*. Our observation of increased methylation within *KCNQ1* at 4/6 CpG sites within 258 bases of each other in TSS1500 (∆beta 0.061-0.104, cg03371125, cg04902871, cg196988309, cg10678459) and two other CpG sites (cg19923326, cg14637411) and *FAP* (1 CpG, ∆beta 0.203, TSS200, cg08826839) before weight loss is consistent with a study of subcutaneous adipose in monozygotic twins; increased methylation in the diabetic twin was reported close to the site of differential methylation we observed for *FAP* at TSS + 86. In *KCNQ1* they reported increased methylation in the diabetic twin of a single CpG site (cg19728223, ∆beta equivalent 0.074) located in the gene body >15 kb away from the closest site identified in our analysis (cg19923326) [39]. We also technically validated our observations for *KCNQ1* by pyrosequencing analysis of the original cohort, and extended this to an additional 12 samples (3 women, 9 men). Interestingly we did not observe significant differential methylation of 2/15 CpG sites interrogated in men (Additional file 6), an observation which may warrant further investigation in a larger cohort with mixed gender. In addition, differential methylation of type-2 diabetes genes in thigh subcutaneous adipose tissue after exercise intervention was recently reported [40]; we observed differential methylation within five of these. Rönn *et al.* reported increased methylation after exercise intervention of 8/10 and 1/6 CpG sites in *KCNQ1* (∆beta 0.015-0.045) and *TCF7L2* (∆beta 0.011-0.046), respectively (with decreased methylation of the other sites) and a single CpG site in each of *BCL11A* (cg01865786, ∆beta 0.03), *FTO* (cg26580413, ∆beta 0.033) and *PROX1* (cg01902845, ∆beta 0.042); these CpG sites were located in the gene body of each loci. Consistent with this we observed hypomethylation of the same CpG site within *FTO* (cg26580413, ∆beta −0.11). For all other CpGs in these genes we observed hypermethylation before weight loss: within *KCNQ1* at 4/6 CpG sites within 258 bases of each other (TSS1500, ∆beta 0.061-0.104, cg03371125, cg04902871, cg196988309, cg10678459) and two other CpG sites (cg19923326, cg14637411); a single site in *TCF7L2* (cg15624624, ∆beta 0.106); two CpG sites within 15 bases of each other in *BCL11A* (cg23556108, cg22445742, ∆beta 0.15, 0.16), and a DMR in *PROX1.* Most of these sites were not located close to those reported by Rönn *et al.*, except the DMR in *PROX1* which was 254 bases away (DMR: Chr1: 214169676–214170679, cg01902845 - Chr1:21470933). As discussed above the similarities and contrasts of the two studies highlight differences in experimental design, the challenges of genome-wide methylation profiling, the complex role of DNA methylation in transcriptional regulation, and the importance of further validatory and in-depth analyses.

A recent study observed and replicated a positive association between DNA methylation in blood at three CpG sites within *HIF3A* (cg22891070, cg27146050 and cg16672562) and BMI [95]. They then analysed data available for subcutaneous adipose tissue and skin, reporting that methylation at cg22891070 was associated with BMI in adipose, but not skin tissue. Consistent with this observation we saw increased methylation at *HIF3A* (cg228910710) before gastric bypass and weight loss (∆beta 0.15, *P* = 3.7 × 10^−8^) in subcutaneous adipose. These observations raise a couple of points. First, that an epigenetic mark associated with BMI and potentially obesity (DNA methylation of cg22891070 in *HIF3A*) may be modifiable, in this case by gastric bypass and or weight loss, and thus, if appropriate, may be tractable to treatment. Second, that blood may have utility as a sample for obesity-related epigenetic biomarker analyses, acting as a surrogate for identifying potentially tissue specific changes of relevance to disease. In this regard, a number of other studies looking at obesity and related traits have profiled global DNA methylation in blood and reported that methylation: co-varies with BMI in blood lymphocytes [25]; shows obesity-related changes in blood leukocytes [26], varies in association with type-2 diabetes [31]; and shows correlations with diet-induced weight loss in peripheral white blood cells [29]. Associations between methylation status in whole blood at birth and body size in childhood have also been reported [96]. We observed an overlap with some of the loci identified in these studies. DNA methylation within *ATP10A* increased (∆beta equivalent 0.025) within a 98 base region of the gene body (Chr15:26026249–26026347) in peripheral blood mononuclear cells from obese or overweight men after an 8-week low-calorie intervention [29]. Consistent with this, we observed increased methylation (∆beta −0.14) of one CpG site in *ATP10A* (cg20049422, Chr15:26044289) in subcutaneous adipose after significant weight loss, although this site is nearly 18 kb from that reported in blood. Variable methylation of *MMP9* has been associated with lean mass in cord blood [96] and with BMI in blood lymphocytes [25]. We observed increased methylation before weight loss (∆beta 0.09) of a single CpG site within *MMP9* (cg04656101, Chr20:44645014) which is consistent with the observation of Feinberg *et al.* [25] who reported a positive correlation between methylation in or near *MMP9* and BMI [25]. In addition, increased methylation of three CpGs in *KCNQ1* (Chr11:2849340–2849503, ∆beta 0.016-0.029) and decreased methylation of one CpG in *FTO* (Chr16:53809231, ∆beta 0.026) was observed in peripheral white blood cells of type-2 diabetics in a case–control study [31]. In line with this, though at some distance from the sites described above, we observed both increased methylation of six CpG sites in *KCNQ1* (4 CpGs Chr16:2464845–2465103, TSS1500, ∆beta 0.061-0.104, two CpG sites Chr: 16:2828778, ∆beta 0.08, Chr16:2858355, ∆beta 0.10) and decreased methylation of *FTO* (∆beta −0.113, Chr16: 54025348) before weight loss. Given the differences between blood and adipose tissue it is tempting to speculate that maybe such similarities reflect a whole body or *intra uterine* response to the obese environment, and/or an inherited epigenetic signature. Also, it arguably provides additional evidence for the utility of blood studies for both the investigation of epigenetic mechanisms at play in obesity, and identification of epigenetic biomarkers of disease; possibilities that warrant further investigation.

Clinical parameters measure changes in the body and blood environment. Tissues exist within this environment, and the epigenome is highly dynamic changing in response to environmental stimuli, such as nutrient availability [19-23,37], and physical exercise [40]. In this context we interrogated changes in DNA methylation at individual CpGs in subcutaneous adipose and clinical trait, and observed correlations passing a robust threshold of absolute R >0.75 and adjusted *P* <0.05 (Benjamini-Hochberg) for sites in 40 genes and at least one clinical measure (Additional file 4). While each correlation is between a single CpG site and a clinical parameter potential significance is suggested by the presence of correlations between genes with biological relevance to the trait. Changes in methylation at single CpGs within *FOXP2* (cg26580413, ∆beta 0.21) and *ACSL1* (cg00287477, ∆beta 0.09) were correlated with changes in weight (*FOXP2* R = 0.85 and *ACSL1* R = 0.91). Copy number variants in *FOXP2* have been associated with childhood obesity [83] and nominally associated with obesity in a familial case–control study [97]. *ACSL1* encodes long-chain acyl CoA synthetase 1 an enzyme which catalyzes thioesterification of fatty acids and knock-down of this gene in 3 T3-L1 adipocytes suppressed lipogenic genes, and increased expression of TNFα and IL6 [98]. A correlation between change in fasting glucose and methylation within *HDAC4* (cg26078407, ∆beta −0.11) was observed (R = −0.75). This is interesting given that HDAC4 is a class II histone deacetylase which is reported to downregulate *GLUT4* transcription in cultured adipocytes and fasting mice [99], and that downregulation of the HDAC4 protein was recently reported in obese subjects with expression induced by exercise [100]. Furthermore, differential methylation and mRNA expression of *HDAC4* was observed in thigh subcutaneous adipose after exercise intervention [40]. The authors also silenced *HDAC4* in 3 T3-L1 adipocytes showing that *HDAC4* repression appeared to result in increased lipogenesis. We also observed a correlation between change in DNA methylation in *SLC37A3* (cg15751131, ∆beta −0.10), a sugar-phosphate exchanger for which an association with congenital hyperinsulinemia has recently been reported [101], and fasting glucose (R = −0.80). Perhaps most intriguingly we observed a correlation between changes in DNA methylation in *DENND1C* (cg11599981, ∆beta 0.12) and both fasting glucose (R = 0.75) and HbA1c (R = 0.85). Little is known about *DENND1C* which is a member of a class of DENN domain Rab GDP-GTP exchange factors (GEFs) [102]. Different GEFs can bind the same Rab protein and appear to modulate Rab activity toward different cellular functions [104]. DENND1C can act as a GEF for Rab35 [103,104], and Rab35 has been shown to play a role in insulin stimulated GLUT4 trafficking in adipocytes [105]; taken with our data this suggests that future investigation of *DENND1C* may be warranted to explore a potential role in glucose transport and homeostasis.

The potential significance of our data is further supported by our observation of significant differential mRNA expression of five genes (*ACACA*, *CTGF*, *S100A8*, *S100A9* and *CETP*) showing differential promoter methylation in subcutaneous adipose (Table 6). All these genes are of potential biological significance: mRNA expression of *ACACA* and *CTGF* in adipose tissue is regulated by caloric intake [67,68] and upregulation of CTGF was observed in subcutaneous adipose in a model of dietary induced obesity [106]; differential expression of *CETP* during weight loss and maintenance was recently reported [69]; and the products of *S100A8* and *S100A9* form the calprotectin complex, a biomarker of obesity, for which increased circulating levels are associated with abdominal obesity, and adipose and systemic tissue inflammation [107-109].

Our study was necessarily constrained by its clinical nature: the sample size, and rare occurrence of repeat operation after gastric bypass, but we believe significant strength is provided by the paired nature of the analyses which reduces the effects of inter-individual variability. Also, gastric bypass achieves major, sustained weight loss and improvement of many obesity co-morbidities [43,44,93,94]; as such we consider it a model akin to an extreme phenotype approach which should increase the chance of identifying biologically relevant changes associated with obesity, its related co-morbidities and weight loss. We believe our data support the validity of this approach as evidenced by our observation of differentially methylated loci passing robust multiple testing adjustment which: are present not only in genes involved in obesity and type-2 diabetes, but loci previously reported to show variable DNA methylation in associated phenotypes; are located in the promoter regions of genes showing differential mRNA expression; show consistent effect over extended regions which supports their potential biological relevance, and show correlations between differential methylation and changes in clinical trait.

## Conclusions

Here we report significant changes in the DNA methylome of two adipose tissue depots (subcutaneous adipose and omentum) after gastric bypass and weight loss. To our knowledge this is the first study to report global DNA methylation profiling of both adipose tissues before and after major weight loss. While it does not allow a distinction between cause and effect with regard to weight loss, it provides additional support of previously reported observations of DNA methylation changes associated with obesity and related traits, suggesting that this mechanism plays a key role in the biology of obesity. It also highlights additional loci with a potential role in disease as good candidates for future analyses. Furthermore, it suggests the possibility that epigenetic tissue remodelling of obesity genes in response to gastric bypass may occur. Thus, the study provides a solid basis for future work and offers further support for the role of adipose tissue DNA methylation in obesity.

## Materials and methods

### Sample inclusion

All subjects gave their written informed consent; The Central Regional Ethics Committee, Wellington, New Zealand approved the study, which complied with the Helsinki Declaration for human research.

The subjects included 15 obese women who underwent gastric bypass by a single surgeon at Wakefield Hospital, Wellington, New Zealand and who returned for a second operation (incisional hernia repair (n = 8), incisional hernia repair and abdominoplasty (n = 4), silastic ring removal (n = 2) or roux loop lengthening (n = 1). Individuals had lost at least 27% of their original body weight (mean 37%, +/− 7.9) by the time of the second operation, representing a mean decrease in BMI of 18.3 +/− 7.8. Mean time between operations was 17.5 months with a range of 9 to 31 months. Clinical and anthropometric data were collected prior to each operation and are summarised in Table 1.

Adipose tissue samples were taken at time of surgery, immediately snap frozen in liquid nitrogen and stored at −80 C.

### DNA extraction

DNA was extracted from approximately 100 mg of tissue using a QIAamp DNeasy Tissue Kit (Qiagen) as per the manufacturer’s protocol, with a 3-h initial lysis step and RNase treatment. DNA was quantitated using a Nanodrop.

### DNA methylation 450 K Illumina BeadChip

Genomic DNA (500 ng) from each sample was treated by bisulphite conversion with the EZ DNA Methylation Kit (Zymo Research Corporation, Irvine, CA, USA) according to manufacturer’s recommendations. A total of 4 μL of bisulphite converted DNA was analyzed using Illumina Infinium Human Methylation 450 K BeadChip Array technology (Illumina, Inc., San Diego, CA, USA) according to the manufacturers protocol. Beadchips were scanned using the Illumina HiScanSQ system that employs a two-colour laser (532 nm/660 nm) fluorescent scanner with a 0.375 μm spatial resolution. The intensities of the images were extracted using Genome Studio (2011.1) Methylation Module (v1.8.5). The Illumina Human Methylation 450 K BeadChip interrogates 485,577 cytosine positions in the human genome; the majority are CpG dinucleotides, with 3,343 CNG sites (throughout the paper these are referred to collectively as CpG sites); 365,934 sites are located within known gene regions (promoter, gene body and UTRs [untranslated regions], and 119,830 are intergenic (see [110]).

### DNA methylation pyrosequencing

Genomic DNA samples were sent to a commercial provider, Exiqon (USA). For genes selected for pyrosequencing all CpG probe sites passing Bonferroni correction were interrogated. For subcutaneous adipose tissue these were: *CETP* (cg03232842), *IFFO1* (cg10838410); *CTGF* (cg00516030, cg17359975); *CMIP* (cg02573873, cg05438708, cg05705335, cg08344351); and *KCNQ1* (cg03371125, cg04902871, cg10678459, cg14637411, cg19698309, cg19923326). For omentum: *PARD3B* (cg12157387); *PLIN4* (cg01907005); *MYO1C* (cg06579248); and *PDE7B* (cg04627183). Depending on the assay, amplicon and sequence content additional CpG sites surrounding the loci of interest were also analysed. Pyrosequencing assays were designed, optimised, performed and analysed by Exiqon with both pyrograms and assay result data supplied.

### RNA extraction

Total RNA was extracted from approximately 100 mg of tissue using Trizol (Invitrogen) as per the manufacturer’s protocol, with an additional chloroform extraction. RNA quantity and quality were assessed using a Nanodrop and Agilent Bioanalyser, respectively. All RNA samples had an RNA integrity number (Agilent Bioanalyser) >7.

### QRTPCR

A total of 500 ng RNA was reversed transcribed using a SuperScript® VILO™ cDNA Synthesis Kit (Life Technologies) following the manufacturer’s instructions. QRTPCR was performed (RocheLight Cycler 480II) using 2 μL 1/5 dilution of cDNA, Perfecta Toughmix (dNature) and TaqMan gene expression assays (Life Technologies). Assays were: CETP (Hs00163942_m1), CTGF (Hs01026927_g1), ACACA (Hs01046047_m1), S100A8 (Hs00374264_g1), S100A9 (Hs00610058_m1) and PLIN4 (Hs00287411_m1). 18 s (4319413E) and PPIA (*cyclophilin A*, 4326316E) were selected as endogenous controls based on previous gene expression studies in adipose tissue [66,67,69,111-113].

### Data analysis

Analyses were performed using R version 2.15.2 [114], Bioconductor [115] packages and custom bash scripts.

### DNA methylation

Each adipose tissue depot, omentum and subcutaneous adipose, was treated independently. Raw intensity data (Illumina 450 K idats) were parsed into the Bioconductor minfi package [116]. Background correction and control normalisation was implemented in minfi. Probes were classed as failed if the intensity for both the methylated and unmethylated probes was <1,000 (based on intensities observed for negative control probes). Any probe which failed in at least one sample, was removed from the entire dataset. All probe sequences were mapped to the human genome (hg19) using BOWTIE2 [117] to identify potential hybridisation issues. 33,457 probes were identified as aligning greater than once and these were removed from the entire dataset. In addition, as our sample cohort was female, Y chromosome probes were removed from the dataset. Due to the paired nature of the samples we chose to retain probes annotated to contain SNPs. Methylation is expressed as a beta value, which is calculated as the intensity of the methylated channel divided by total intensity ((methylated + unmethylated) + 100). Beta-values range between 0 (unmethylated) and 1 (completely methylated) and can be interpreted broadly as the percentage of methylation for a particular CpG. Methylation for genomic regions was calculated as the mean beta for all probes located within the region as annotated by Illumina, TSS200 (TSS - transcription start site), TSS1500, 5′UTR (UTR - untranslated region), 1st Exon, gene body, 3′UTR and intergenic. A paired t-test was used to test for statistical difference between the means within each tissue independently (subcutaneous adipose and omentum) before and after weight loss. Global methylation profiling of 15 different normal tissue samples (adipose, bladder, blood, brain, breast, bone, eye, heart, kidney, liver, lung, prostate, skin, stomach, tongue) used publically available datasets catalogued by the R package Marmal-aid [118].

Mean ∆beta was calculated for a given CpG site before weight loss (at gastric bypass) compared to after weight loss (at second operation). Throughout the manuscript mean ∆beta is referred to as ∆beta. A positive ∆beta indicates relative hypermethylation before weight loss, and a negative ∆beta relative hypomethylation. Total ∆beta was distributed normally for both subcutaneous adipose and omentum (data not shown). Given the paired nature of the samples a paired samples t-test was used to test for significance. Corrections for multiple testing were made using a Bonferroni adjustment for all sites passing quality filtering (as above) [47]. All ∆beta values and the related differentially methylated CpG sites mentioned in the text have passed Bonferroni correction unless stated otherwise. Divisive hierarchical clustering was performed using the Bioconductor Cluster package [119], and the diana algorithm. Probes were annotated using Illumina450K Manifest information. The Illumina 450 K data has been deposited in the EBI ArrayExpress database (E-MTAB-3052).

Identification of DMRs was performed using the probe lasso method implemented in the R package ChAMP [48]. This method uses a feature based dynamic window incorporating probe association statistics (*P* values) and genomic feature annotation (probe distribution) to identify regions of differential methylation between two groups, in this case before and after gastric bypass and weight loss. We set a lasso radius of 2,000 bases, the number of minimum significant probes in the lasso was three, and the minimum DMR separation was 1,000 bases. Individual probes had to show an adjusted *P* value of 0.5 (Benjamini-Hochberg) to be incorporated in to the analysis. Reported DMRs passed a statistical threshold of *P* = 0.05).

### Pathways enrichment

Enrichment analysis was performed using the WebGestalt (WEB-based Gene SeT AnaLysis) Toolkit [120], [121]. IDs were uploaded and analysis performed against the human reference genome using a Bonferroni multiple test adjustment threshold of *P* <0.05.

### QRTPCR

The mean expression (Ct) for 18 s and *PPIA* combined was used to normalise the expression of each target gene (∆Ct). Differential expression before and after gastric bypass and weight loss was assessed using a paired t-test. Fold change in expression between the two time points (before/after weight loss) was calculated using 2^-∆∆Ct^.

## Additional files

Additional file 1:
**Global methylation profile of subcutaneous abdominal adipose and omentum (as Figure** **1** **) alongside 15 different healthy tissues.** Mean methylation was calculated across all probes within each gene region (TSS200, TSS1500, 5′UTR, 1st exon, gene body, 3′UTR, intergenic) annotated as the Illumina 450 K manifest for 15 tissues (adipose, bladder, blood, brain, breast, bone, eye, heart, kidney, liver, lung, prostate, skin, stomach, tongue). Mean beta is plotted on the Y axis, and errors bars denote +/− standard deviation. * Significant difference between average DNA methylation before and after weight loss, Bonferroni adjusted *P* <0.05. Publically available data for healthy tissues was obtained through catalogues provided by the R package Marmal-aid. Additional file 2:
**Annotated data for the 3,601 differentially methylated CpG sites passing Bonferroni correction in the analysis of subcutaneous adipose before and after weight loss.** Illumina probe ID, probe type, gene name and accession number (as Illumina 450 K manifest) are shown along with methylation statistics; mean_beta_before and mean_beta_after present mean beta values before and after weight loss, respectively. Additional file 3:
**List of DMRs mapping to 162 annotated loci identified in subcutaneous adipose tissue.** Location, size, *P* value, and basic annotation are given for each DMR. Additional file 4:
**Correlations between changes in DNA methylation and clinical trait in subcutaneous adipose.** Data for probes mapping to annotated genes which showed a Pearson’s correlation between ∆methylation and ∆clinical trait of absolute R >0.75 and *P* <0.05 (Benjamini Hochberg adjusted) for at least one trait are shown. Additional file 5:
**Correlations for the comparison of 450 K array data with pyrosequence analysis for 15 CpG sites in the subcutaneous adipose samples.** Illumina 450 K beta values are plotted (Y axis) against percent methylation using pyrosequencing (X axis). R2 and *P* values are shown. Additional file 6:
**DNA methylation data obtained from the pyrosequence analysis of subcutaneous adipose in 18 women and nine men before and after weight loss.** Statistics for methylation before and after weight loss and gastric bypass (mean_methylation_before, mean_methylation_after) are presented. A paired t-test was performed to test for a significant difference in methylation before and after weight loss. CpG sites interrogated (CpG(pyro)) are represented as, for instance, ‘CpGd122’ indicating their location in relation to all other CpGs either ‘u’ upstream or ‘d’ downstream from the transcription start site (TSS) (for example, CpGd122 is CpG number 122 downstream of the TSS). Chromosomal locations and Illumina probe IDs are shown. Additional file 7:
**DNA methylation data obtained from the pyrosequence analysis of omentum in 17 women and five men before and after weight loss.** Statistics for methylation before and after weight loss and gastric bypass (mean_methylation_before, mean_methylation_after) are presented. A paired t-test was performed to test for a significant difference in methylation before and after weight loss. CpG sites interrogated (CpG(pyro)) are represented as, for instance, ‘CpGd122’ indicating their location in relation to all other CpGs either ‘u’ upstream or ‘d’ downstream from the transcription start site (TSS) (for example, CpGd122 is CpG number 122 downstream of the TSS). Chromosomal locations are also presented as are Illumina probe IDs.

## Abbreviations

BMI: Body mass index
CpG: Cytosine guanine dinucleotide
DMR: Differentially methylated region
GO: Gene ontology
GWAS: Genome-wide association study
HbA1c: Glycated haemoglobin
HDL: High density lipoprotein
LDL: Low density lipoprotein
SNP: Single-nucleotide polymorphism
TSS: Transcription start site
UTR: Untranslated region. Where a genomic location is specified in text, for example, Chr2,162929997-16292979 co-ordinates are as per genome build 37
