# Supplementary figures and images for: An analysis of DNA methylation in human adipose tissue reveals differential modification of obesity genes before and after gastric bypass and weight loss
Source: Genome Biol. 2015 Jan 22;16(1):8. doi: 10.1186/s13059-014-0569-x (PMC4301800; doi:10.1186/s13059-014-0569-x)

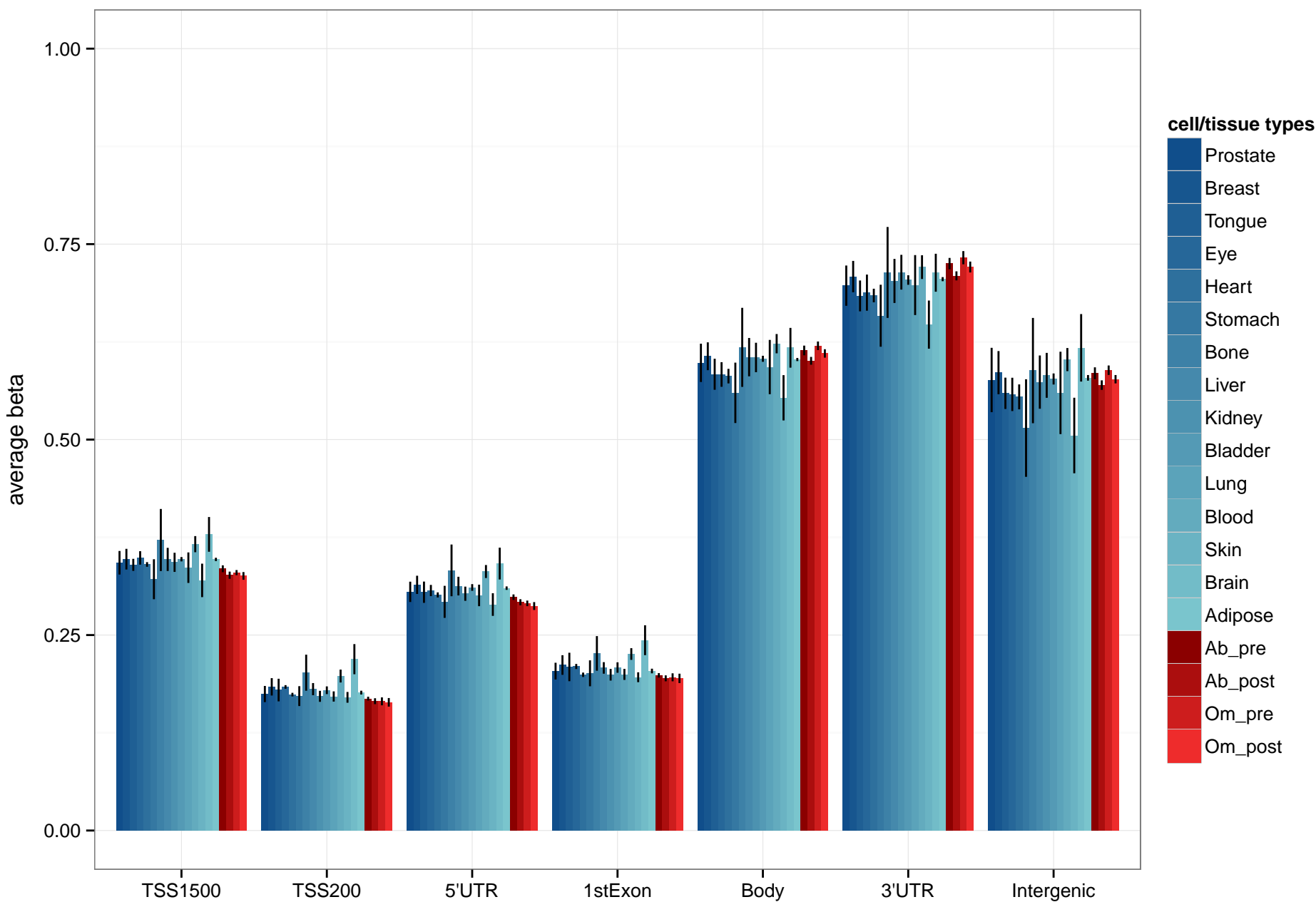

Supplement: Additional file 1: — Global methylation profile of subcutaneous abdominal adipose and omentum (as Figure 1 ) alongside 15 different healthy tissues. Mean methylation was calculated across all probes within each gene region (TSS200, TSS1500, 5′UTR, 1st exon, gene body, 3′UTR, intergenic) annotated as the Illumina 450 K manifest for 15 tissues (adipose, bladder, blood, brain, breast, bone, eye, heart, kidney, liver, lung, prostate, skin, stomach, tongue). Mean beta is plotted on the Y axis, and errors bars denote +/− standard deviation. * Significant difference between average DNA methylation before and after weight loss, Bonferroni adjusted P <0.05. Publically available data for healthy tissues was obtained through catalogues provided by the R package Marmal-aid. [file 13059_2014_569_MOESM1_ESM.pdf]

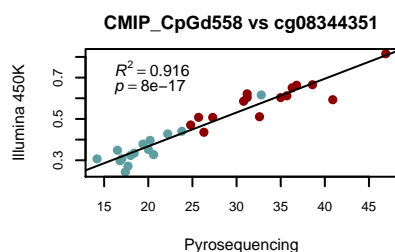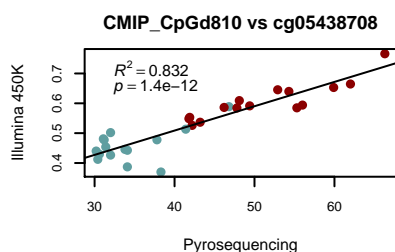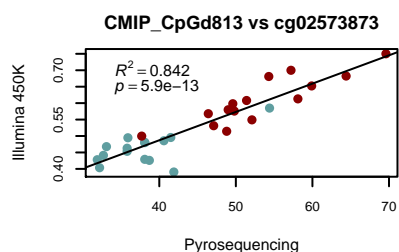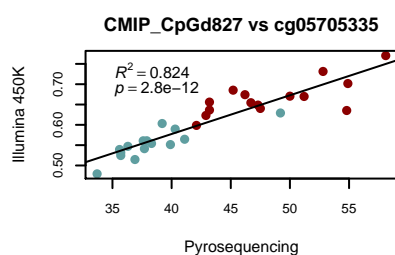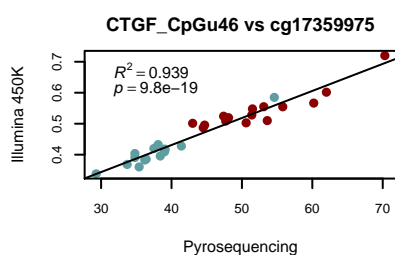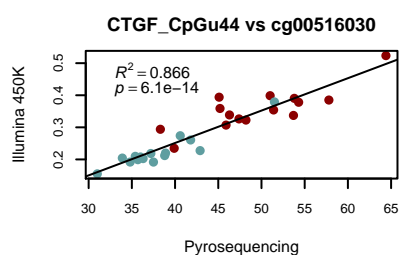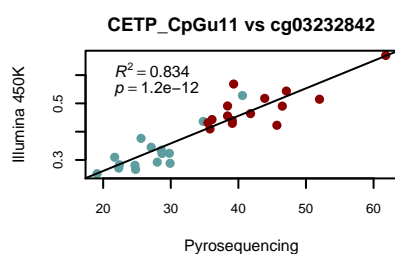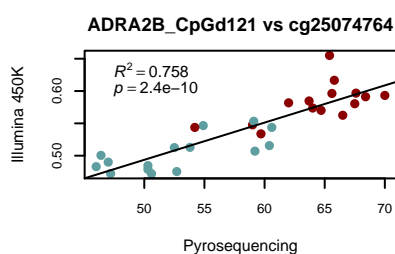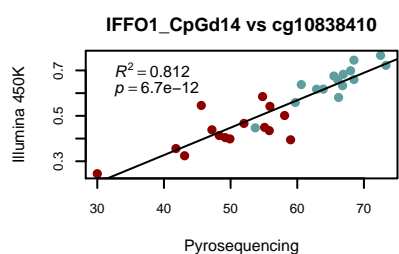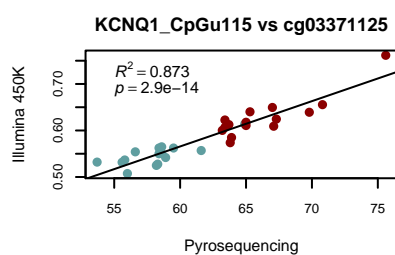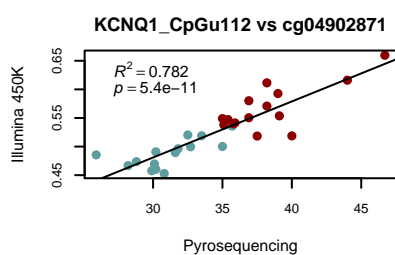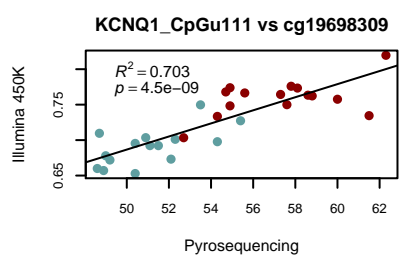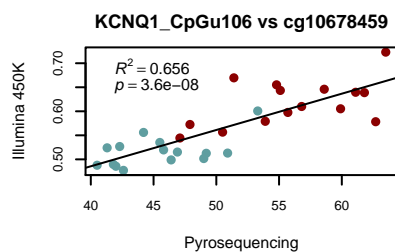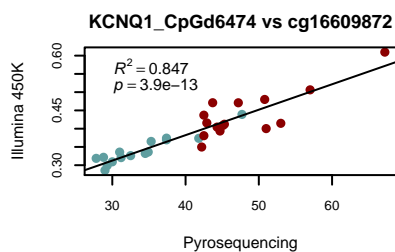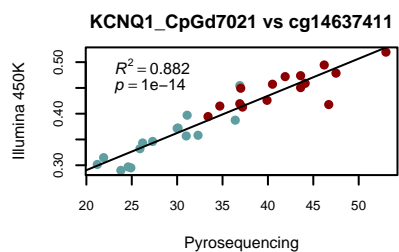

Supplement: Additional file 5: — Correlations for the comparison of 450 K array data with pyrosequence analysis for 15 CpG sites in the subcutaneous adipose samples. Illumina 450 K beta values are plotted (Y axis) against percent methylation using pyrosequencing (X axis). R2 and P values are shown. [file 13059_2014_569_MOESM5_ESM.pdf]
